# Evaluation of a New Approach for Modeling Full Ring Stent Bundles with the Inclusion of Manufacturing Strains

**DOI:** 10.1007/s10439-019-02322-0

**Published:** 2019-07-17

**Authors:** Faidon Kyriakou, David Bow, William Dempster, Robbie Brodie, David Nash

**Affiliations:** 1 Department of Mechanical and Aerospace Engineering, University of Strathclyde, Glasgow, G1 1XJ UK; 2 MedAlliance, Glasgow, G52 4GA UK; 3 Terumo Aortic, Inchinnan, Glasgow, PA4 9RR UK

**Keywords:** Stent, Finite element analysis, Aneurysm, Ring bundle, Anaconda

## Abstract

Ring stent bundles have been used in several biomedical stent-graft devices for decades, yet in the published literature, the numerical models of these structures always present significant simplifications. In this paper, a finite element (FE) ring stent bundle has been developed and evaluated with a combination of beam and surface elements. With this approach, the shape, the global stiffness and the strains of the structure can all be well predicted at a low computational cost while the approach is suitable for application to non-symmetrical, patient-specific implant simulations. The model has been validated against analytical and experimental data showing that the manufacturing strains can be predicted to a 0.1% accuracy and the structural stiffness with 0–7% precision. The model has also been compared with a more computationally expensive FE model of higher fidelity, revealing a discrepancy of 0–5% of the strain value. Finally, it has been shown that the exclusion of the manufacturing process from the simulation, a technique used in the literature, quadruples the analysis error. This is the first model that can capture the mechanical state of a full ring stent bundle, suitable for complex implant geometry simulations, with such accuracy.

## Introduction

**Figure 1:**
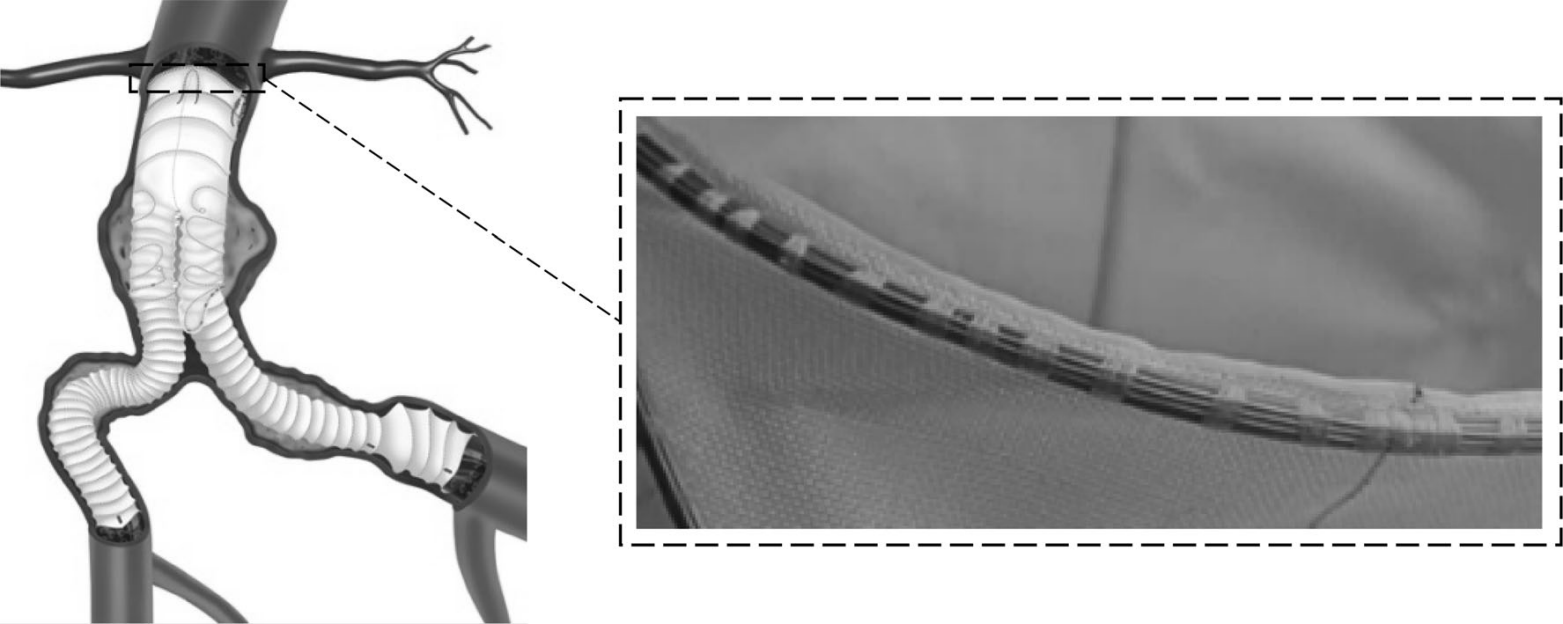
The Anaconda™ stent graft placed inside an abdominal aortic aneurysm (AAA).^25^ The 1st proximal ring bundle is shown in detail illustrating the multiple turns of Nitinol wire sutured onto the fabric.

Stent grafts are medical devices used for the treatment of aneurysms; the irreversible dilation of blood vessels by at least 50%. With the use of catheters, these devices are inserted and deployed into large aneurysmal arteries, usually the aorta or the iliacs (Fig. 1). During the last decade, computational models have been developed to support the design of stent grafts, in order to better understand the relation between device geometry and mechanical variables of medical importance (e.g., maximum strain, radial force, fixation force),^2^,^10^,^14^,^15^,^20^–^22^ providing insights for both clinicians and stent device manufacturers alike.

Most numerical studies, however, have been conducted on the more common Z-shaped stents, since these geometries are the most dominant in the market. The behavior of alternative geometries has rarely been examined. An interesting exception is the work of Demanget *et al*.^8^ where eight commercially available stent grafts were tested under bending and pressurization, underlining the effect of the global design of these devices on their mechanical performance.

Herein, ring stent bundles are considered. Such geometries are found in devices like the Anaconda™ (Terumo Aortic, Glasgow, UK) and so far, whenever numerically examined, researchers employ significant simplifications to study them mechanically. The reduction of the number of wire turns,^8^,^23^,^24^ the omission of the manufacturing process^8^,^23^,^24^ and the utilization of double symmetry^4^,^26^ are the major modeling techniques followed, all restricting the models’ accuracy or applicability. As a result, to the best of the authors’ knowledge, there is no model currently available in the literature that can capture the mechanical state of a full ring stent bundle accurately, with the versatility to be applied to complex implant geometry simulations.

In the present study, the development and evaluation of a reliable finite element analysis (FEA) model of a full ring bundle consisting of Nitinol wires was pursued. This model did not use symmetry conditions while it did take into account the strains induced in the structure due to the manufacturing process. At the same time, computational expense was kept at a minimum. The FEA model presented constitutes a new approach on ring bundle analysis, since the developed technique allows the reliable simulation of full ring models. This approach can permit the reliable assessment of strains, can enable the deployment in asymmetric and/or patient-specific conditions and can pave the way for efficient full device models. Such abilities can enhance the current understanding of ring stent failures and aid future stent designs in accommodating challenging aneurysmal cases, eventually reducing endoleaking and migration, two of the major causes of post-op endovascular aneurysm repair (EVAR) complications.^18^,^27^

## Materials and Methods

**Figure 2:**
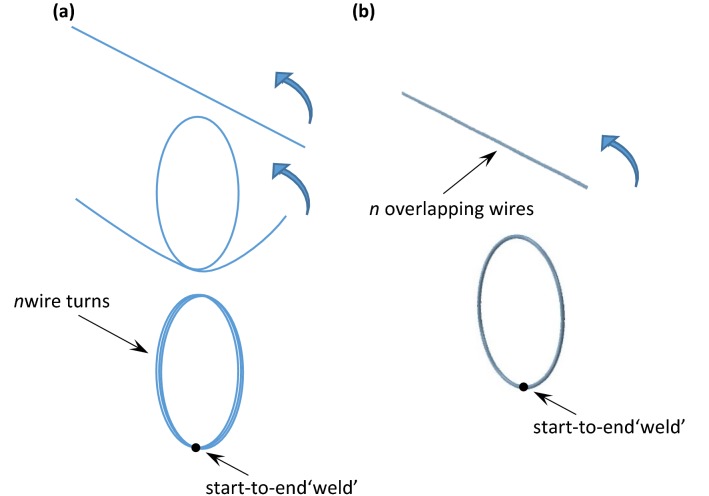
During manufacturing, a wire is turned *n* times to create a bundle (a); yet herein, *n* wires that occupy the same space are turned once (b).

Ring stents present in devices such as the Anaconda™ are circular wire bundles sutured to a tubular polyester fabric (Fig. 1). Each one of these bundles is constructed from multiple turns of a single Nitinol wire. The wire is originally straight and is formed into a bundle by being turned multiple times onto a mandrel; then, its two ends are crimped to form a closed ring (Fig. 2a). Because the ring stents act as discrete components, with the fabric accommodating the curves of the artery, it has been deemed appropriate to simplify the complex loading environment the endovascular ring stent device experiences to focus on radial loading of a discrete ring stent. Herein, Abaqus/Standard (version 6.13-2, Dassault Systemes Simulia Corp., RI, USA) has been used to model a ring stent under two case studies, a load–deflection test and a deployment inside a vascular section. The modeling approach followed is presented below.

### The Beam Full Ring Model (BF Model)

To model an *n*-turn ring bundle, *n* straight superimposed wires of Timoshenko beam elements (B32H) were formed into a circle and subsequently joined start-to-end. In other words, instead of turning 1 Nitinol wire *n* times (as in manufacturing), *n* overlapping (i.e., occupying the same space) wires were considered, joined together so that the bundle would behave as a single entity, and then turned once, in the first step of the simulation (Fig. 2b). After that, the *n* wires were tied start-to-end with a ‘weld’ constraint to remain circular. Because the whole bundle behaves as one, turning it one time is, generally speaking, *n* times faster than turning 1 wire *n* times. Similarly, boundary conditions need only to be applied to a single wire of the bundle, a useful feature for the reduction of computational cost.

Although beam elements can capture the mechanical response of the ring bundle, they cannot fully represent its cross-sectional geometry, especially because of the wire superposition. This aspect is important since correct topological representation means that once the ring is deployed into the artery, it will be correctly deformed and hence, acquire an accurate global shape which will lead to an accurate strain state.

**Figure 3:**
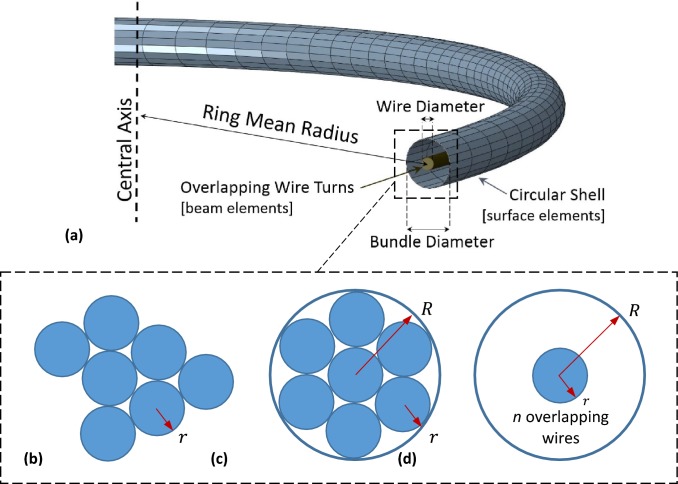
Section of the BF model. *n* superimposed wire turns are enclosed inside a shell that represents the bundle (a). For the identification of the bundle diameter, an approximation is used. More specifically, the cross-section of any realistic bundle configuration (b) is reconfigured according to the circle packing theory (c) and the minimum circle, *R*, that can enclose the wire turns is calculated. The BF model’s cross-section (d) has a bundle diameter, *R* and hosts all the turns overlapped at its center. All the wire turns have radius, *r*, as in the original configuration.

For that reason, surface elements (SFM3D4R) were used to create a circular shell representing the bundle surface (Fig. 3a). These elements do not add any stiffness to the structure; however, by tying them to the beam elements at the beginning of the analysis, they can provide the model with a close approximation of the bundle size. Nevertheless, the identification of the appropriate bundle diameter needs some consideration.

In reality, the cross-section of the bundle consists of tightly arranged wires that stay in place due to sutures. These ties, though, are manually created during manufacturing and result in the inconsistent arrangement of the Nitinol turns through the circumference of the rings. As a result, there is no straightforward way to calculate the diameter of each bundle. In order to acquire an estimator of the cross-sectional diameter of the bundles, results from circle packing theory were utilized. More specifically, the results regarding arranging a specific number of circles (in this case, Nitinol turns) inside the smallest possible circle (in this case, bundle approximation) were used^5^,^12^ (Figs. 3b–3d). The geometry configurations studied are reported in Table 1 and use the ratio of the bundle radius to the wire radius (BW) as an indicator of the overall geometry. For *n* wire turns (*n *= 1, …, 14) the bundle will sequentially increase in strength. By combining beam and surface type elements, the BF model that closely approximates both the stiffness and the topology of the bundle was created (Fig. 3a). Moreover, due to the superposition of Nitinol turns, neither case-specific cross-sectional arrangements needed to be modeled, nor wire-to-wire contact interactions, keeping both the modeling complexity and the computational expense to a minimum.

**Table 1:** The ratio *BW* allows the construction of a bundle with the smallest possible cross-section that can accommodate *n* wires of a given radius.

*n*	$BW = \frac{{R_{\text{bundle}} }}{{R_{\text{wire}} }}$	*n*	$BW = \frac{{R_{\text{bundle}} }}{{R_{\text{wire}} }}$
1	1.000	8	3.304
2	2.000	9	3.613
3	2.154	10	3.813
4	2.414	11	3.923
5	2.701	12	4.029
6	3.000	13	4.236
7	3.000	14	4.328

The initial ring formation was assessed analytically, while two case studies were investigated further: a load-deflection test based on a laboratory experiment; and avascular deployment, compared with a higher fidelity FEA model. For both cases, four rings were used as representative of the wide range of possible wire and ring diameter values available in clinical practice (Table 2).

**Table 2:** Configuration of the 4 ring bundles used (see Fig. 3a, for the variable definitions).

Variables	Ring 1	Ring 2	Ring 3	Ring 4
Wire diameter (mm)	0.180	0.160	0.220	0.200
Ring mean diameter (mm)	27.02	33.16	39.25	48.09
Number of turns	10	8	14	9
Bundle diameter (mm)	0.69	0.53	0.95	0.72

For simulating the Nitinol alloy, a user defined subroutine (UMAT) that follows the constitutive model proposed by Auricchio^1^ was used. The density was set to 6.45 g/cm^3^ and the material parameters employed were calibrated specifically for the Nitinol wires at 37 °C by Boukis^3^ (Table 3). Finally, a mesh convergence study ensured the independence of the results to the size of the elements.

**Table 3:** Parameters for the constitutive model of Nitinol.

Austenite elasticity, *E*_A_ (GPa)	59
Austenite Poisson’s ratio, *ν*_A_	0.33
Martensite elasticity, *E*_M_ (GPa)	26.5
Martensite Poisson’s ratio, *ν*_M_	0.33
Transformation strain, *ε*^L^ (MPa)	0.05
Start of transformation loading, *σ*_L_^S^ (MPa)	636
End of transformation loading, *σ*_L_^E^ (MPa)	740
Start of transformation unloading, *σ*_U_^S^ (MPa)	430
End of transformation unloading, *σ*_U_^E^ (MPa)	302
Start of transformation stress in compression (MPa)	965

### Analytical Validation

Due to the manufacturing process of bending the straight wire into a circle, pre-strains are present even in the initial configuration of the manufactured ring stent. As a result, one major objective of the current work was to correctly represent those effects by the inclusion of the manufacturing process in the simulation. This process was analytically validated by comparing the maximum strain *ε* of the four ring bundles at the end of the circular forming phase with the analytical values from classical mechanics: from simple beam bending theory the maximum strain at the outer surface of a round beam can be given by $\varepsilon = R_{\text{wire}} /R_{\text{ring}} ,$ where $R_{\text{wire}}$ and $R_{\text{ring}}$ are radii of the wire and ring respectively.

### ‘Saddle Pull Test’ Validation

**Figure 4:**
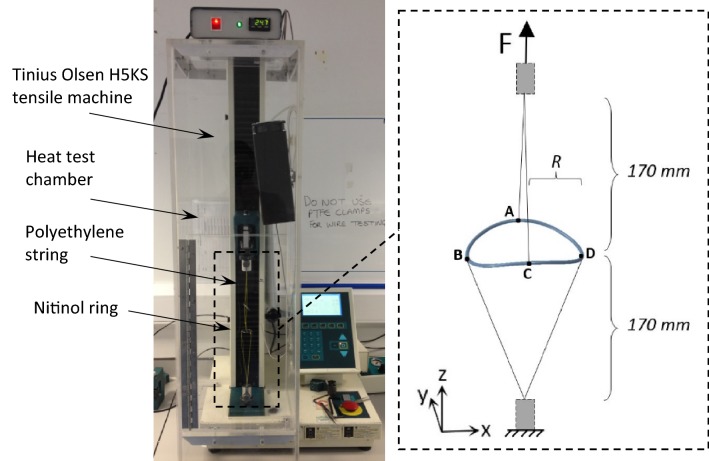
The experimental set-up of the ‘saddle pull test’. In the schematic, pairs A, C and B, D represent the peaks and valleys of the ring bundle respectively.

In order to compare the stiffness of the BF model with experimental results, a load-deflection set-up was employed. More specifically, the ‘saddle pull test’ used is a load-deflection validation which compares the structural stiffness and load exerted by the ring stent over a range of superelastic deflections which are representative of *in-vivo* deformations. Herein, four physical rings with the same specifications as the ones outlined in Table 2, were tied at four equidistant points along their circumference and were loaded in such a manner that the deformed shape *in vivo*, termed ‘saddle shape’, was replicated (Fig. 4).

During the experiment, each bundle was attached to four polyethylene strings with an average stiffness of 7.41 N/mm per 100 mm length. The strings were connected to a Tinius Olsen H5KS tensile testing machine with a 50 N load cell calibrated in the range of 0.5–50 N. The force accuracy was ± 0.5% of the applied load and the displacement accuracy was in the range of ± 0.002 mm. After preconditioning, the bundle was pulled to an extreme (to simulate the strains developed during stent compaction) before being partially unloaded and cycled for 100 cycles, to ensure that the loading-unloading path of the Nitinol ring was settled. Cycling occurred between a position that correlates to high *in-vivo* deflection and a position that correlates to low *in-vivo* deflection. These extreme positions correspond to the saddle shape each ring would result in, if deployed in a 30% or 10% oversize ratio respectively (extremes of the operational range of the device), where

1 $${\text{oversize}} = \left( {\frac{{R_{\text{ring}} }}{{R_{\text{vessel}} }} - 1} \right) \cdot 100\% .$$

Cycling between these positions is extreme and was considered for the purposes of the validation alone. In reality, the motion range of the ring is smaller, as the oversize is fixed and the extremes of its saddle shape are dictated by the motion of the vessel between systolic and diastolic pressure, a range much smaller than the one considered. Every bundle configuration was tested with three samples at 37 °C.

This test method was replicated in Abaqus for the conduction of the validation. Each string was simulated with an Axial Connector of the same stiffness, a special type of element that provides a connection between a pair of nodes without restricting any component of relative motion. All strings were attached to one turn of the bundle while two reference points served as the fixing point and the load cell of the uniaxial tester. The peaks and valleys of the bundles were fully restricted rotationally considering that the strings used to pull the bundles, along with the sutures and the wire to wire interactions, greatly restrained the wire’s rotation. Although, in reality, the tangential restriction (Rotation_x_ = 0 for points A, C and Rotation_y_ = 0 for points B, D—see Fig. 4 for locations) may not be equal to zero, a value close to zero is reasonable to assume. Through the FEA results, force–displacement data were acquired to perform the validation.

### The Manufacturing Process Effect

In order to quantify the effect the manufacturing process has on the global stiffness of the ring bundle, a second set of FE ring models that did not account for the manufacturing strains was compared against the experimental results. Repeating the previous analysis while excluding pre-strains (the rings were not created from straight wires but instead, were directly created as circles) allowed the importance of this modeling step to be illustrated.

### Vascular Deployment

**Figure 5:**
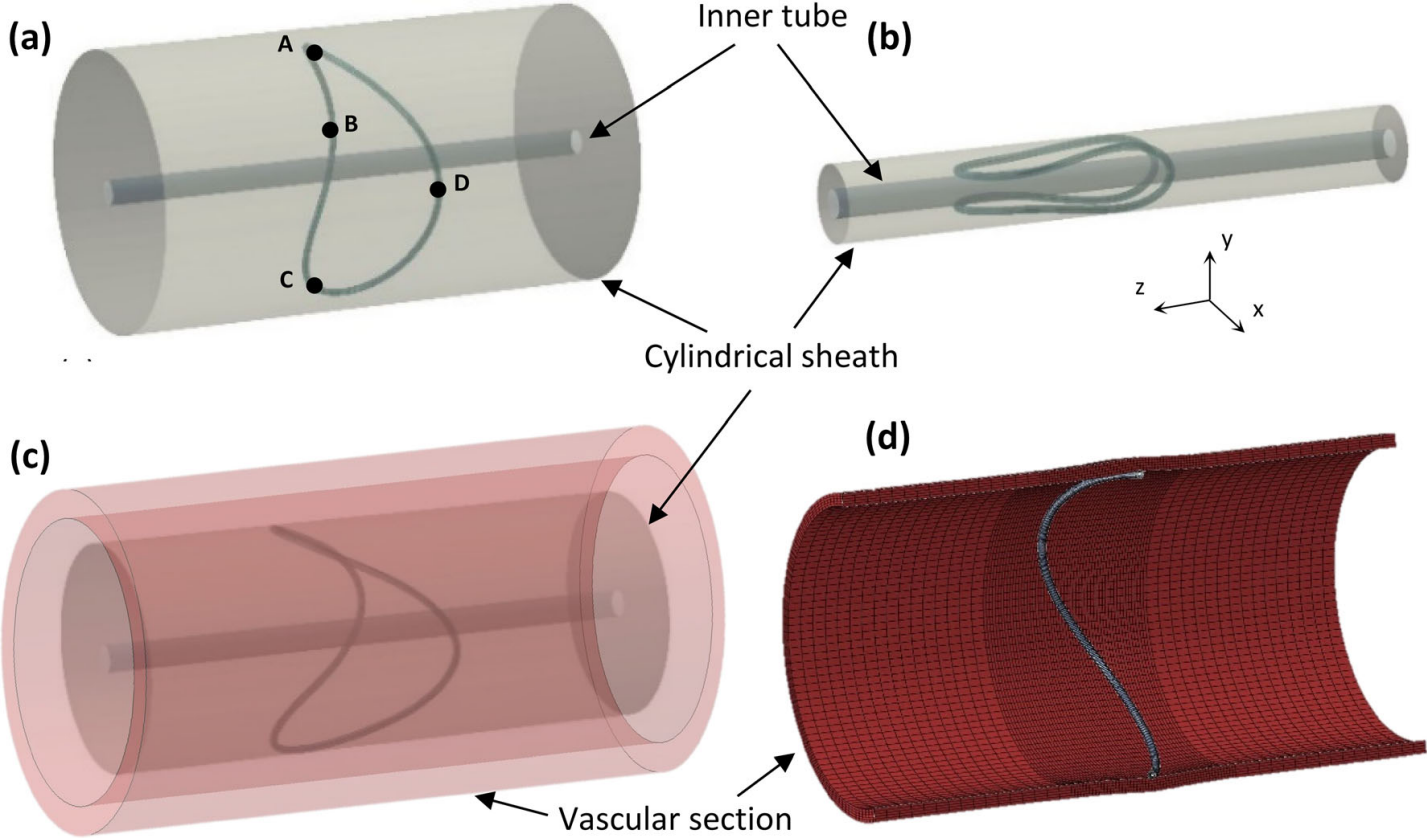
After ring formation, the ring was compacted with the help of a cylindrical sheath (a) down to its delivery size (b). Subsequently, the sheath was inflated (c), allowing the final deployment of the ring inside the vessel, which pulsated between the diastolic and systolic pressure (d). The internal cylindrical surface represents the inner tube present in the physical delivery system.

The second case study conducted involved the deployment of the BF ring stent inside a vascular section. For this purpose, the ring stent was compacted into the clinically accurate catheter size with the use of a cylindrical sheath (SFM3D4R elements) of varying diameter which served as a compacting tool (Figs. 5a and 5b). Furthermore, an internal cylindrical surface was modeled (as a rigid surface) representing the inner tube present in the physical delivery system. Modeling the full compaction of the ring is necessary because of the load-history dependent nature of Nitinol’s stress/strain state. If excluded, the final mechanical state of the material will not capture the *in vivo* state of the device.

After compaction, the ring bundle was subsequently deployed into a vascular segment (Fig. 5c). The deployment occurred *via* the inflation of the cylindrical sheath and the boundary conditions for both compaction and deployment were:

2 $$\left. {\begin{array}{*{20}l} { \bullet \,{\text{Rotation}}_{x} = 0\,{\text{for}}\,{\text{points}}\,{\text{B,D}}} \\ { \bullet \,{\text{Rotation}}_{y} = 0\,{\text{for}}\,{\text{points}}\,{\text{A,C}}} \\ { \bullet \,{\text{Rotation}}_{z} = 0\,{\text{for}}\,{\text{points}}\,{\text{A,B,C,D}}} \\ { \bullet \,{\text{Displacement}}_{x} = 0\,{\text{for}}\,{\text{points}}\,{\text{A,C}}} \\ { \bullet \,{\text{Displacement}}_{y} = 0\,{\text{for}}\,{\text{points}}\,{\text{B,D}}} \\ { \bullet \,{\text{Displacement}}_{z} = 0\,{\text{for}}\,{\text{points}}\,{\text{B}},{\text{D}}} \\ \end{array} } \right\}$$

with points identified in Fig. 5a. These conditions are straightforward in order to secure the stability of the ring. After deployment, the vessel was pressurized between a diastolic pressure, *P*_d_ = 80 mmHg and a systolic pressure, *P*_s_ = 120 mmHg (Fig. 5d). For those steps, in addition to Eqs. (2), the following restriction was also applied:

3 $$\bullet \,{\text{Rotation}}_{y} = 0\;{\text{for}}\;{\text{point}}\;{\text{B}}$$

This condition secured that the ring would not experience a “full body rotation” around its tangential axis while it enhanced the ring/vessel contact and increased the stability of the interaction. The contact between the vessel and the bundle was modeled with a friction coefficient of 0.05, while the contact between the bundle and the catheter was modeled as frictionless.

### The Continuum Quarter Ring Model (CQ Model)

**Figure 6:**
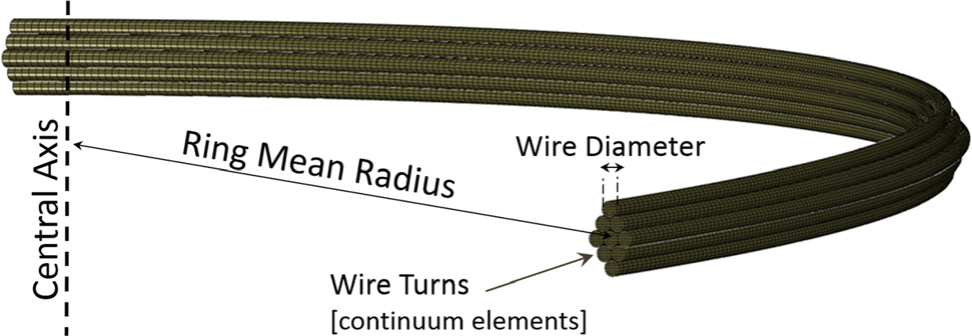
The CQ model. A quarter of the ring stent is modeled with continuum elements. Each wire turn is considered separately.

For the evaluation of the results of the Vascular Deployment, the analysis was repeated with a Continuum Quarter Ring Model (CQ model) of higher fidelity, developed earlier^4^ (Fig. 6). Briefly, this model used double symmetry to describe the ring (hence regarded a quarter section of it), while the Nitinol turns were distributed over the cross-section of the bundle. This led to the wire turns having different initial lengths with each other and different curvatures during the analysis, allowing the model to pick up various wire strains within every cross-section. Other dissimilarities between the models include differences in the contact formulation against the vessel and necessary boundary restrictions for stability. Apart from that, the BF and the CQ models were set up to simulate the four rings in full compaction, deployment and pulsation under the same conditions.

Nevertheless, because the CQ model distributes the wire turns in space, modeling of multi-turn rings brings more variability to the comparison than the mere differences of the finite elements in use. For a direct comparison, a further study was conducted by implementing single turn versions of both modeling methods (i.e., 1 turn of a certain wire diameter for the CQ model and 1 turn of the same wire with surface elements that represents the diameter of 1 turn for the BF model).

Hence, the multi-turn version of each ring corresponded to the real bundle and the 1-turn version served as a 1 on 1 comparison of the models. Note that the 1-turn version of Ring 4 was severely slender and unstable, hence it was excluded from the analysis.

For the comparison, the variables of interest were the chronic outward force (COF, being the sum of the radial component of all (nodal) forces produced by the ring on the vascular surface) and the maximum strains developed in the wire during diastole $(\varepsilon_{\text{Diast}} )$ and systole $(\varepsilon_{\text{Syst}} )$. The maximum mean strain for any location of the ring $({M}\varepsilon )$ was also recorded.

### The Vascular Constitutive Model

All Vascular Deployment analyses used an arterial model developed in Ref. 16. The constitutive equation for this homogeneous, isotropic, phenomenological model was a 6th order reduced polynomial strain energy function:

4 $$U = \mathop \sum \limits_{i = 1}^{6} c_{i} (\bar{I}_{1} - 3)^{i} + \mathop \sum \limits_{i = 1}^{6} \frac{1}{{D_{i} }}(J^{\text{el}} - 1)^{2i} ,$$

where $\bar{I}_{1}$ is the first distortional strain invariant, $J^{el}$ is the elastic volume ratio, defined as the total volume ratio (current volume/original volume) over the thermal volume ratio (herein = 1) and *D*_*i*_ are material parameters. The model was assumed almost incompressible with Poisson’s ratio being over 0.4999 (leading to *D*_1_ = 0.004593 and *D*_*i*_ = 0 for *i* = 2, …, 6) and density = 1.16 × 10^−9^ tonne/mm^3^.

The model was developed to fit the pressure-radius data of Labrosse *et al*.^17^ for males aged 67–77 (Table 4). The vascular sections were straight with length twice the respective ring diameter and thickness 2 mm, while their radius was chosen to produce 10% ring stent oversize at the time-weighted arterial blood pressure *P*_m_ = 93.3 mmHg, defined as:

5 $$P_{\text{m}} = P_{\text{d}} + \frac{1}{3}\left( {P_{\text{s}} - P_{\text{d}} } \right).$$

Finally, it should be noted that all simulations (of the ‘saddle pull test’ and the vascular deployment alike) were run on 4 Xeon© CPUs of a desktop computer (3.40 GHz, 64 GB).

**Table 4:** Fitted coefficients for the 6th order reduced polynomial strain energy function.

*c*_1_	*c*_2_	*c*_3_	*c*_4_	*c*_5_	*c*_6_
0.0048	0.0911	− 1.0600	9.5292	− 31.7421	46.3921

*c*_*i*_ are reported in MPa

## Results

### Analytical Validation

After bending the straight wires into a circular ring, the magnitude of the maximum strains was found to be significant, generally in the order of 0.4–0.7 × 10^−2^ while the FEA model predictions varied less than 0.1% from the analytical solution of $R_{\text{wire}} /R_{\text{ring}}$ for all four rings. This response offers great confidence regarding the accuracy of the initial load step in capturing the manufacturing strains.

### ‘Saddle Pull Test’ Validation

**Figure 7:**
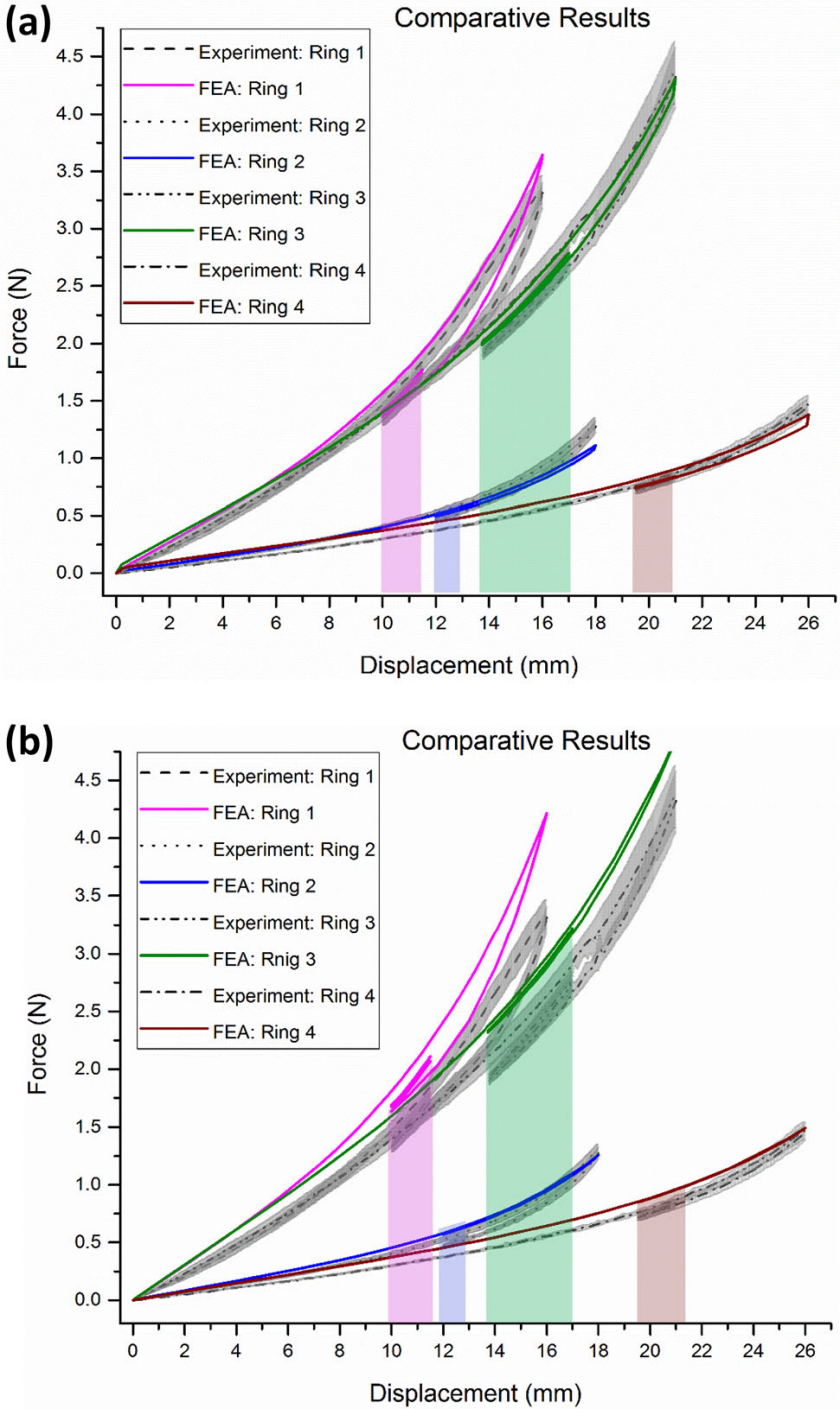
Comparative results of all four ring configurations tested in the ‘saddle pull test’. The gray area corresponds to the standard deviation of the experiments while the coloured regions represent the regions of interest (operational range of motion) for each ring bundle. The model with (a) and without (b) the manufacturing strains is assessed.

Regarding the ‘saddle pull test’, the computational results of the force–displacement curves from the BF model simulations follow the trend of the experimental graphs (Fig. 7a) and at the region of interest (i.e., the cycling phase of the loading which corresponds to the *in vivo* conditions of the stent graft inside the aorta) are in good agreement with the experimental values. Discrepancy of the FEA analysis when compared to the experimental load cell measurements at the cycling region for each bundle is reported in Table 5.

**Table 5:** Deviation of the FEA force–displacement response from the corresponding experimental results for each ring bundle at its operational region.

Ring	FEA deviation in force from the experimental mean values (%)
Ring 1	3.2–5.4
Ring 2	1.5–7.0
Ring 3	1.2–3.3
Ring 4	0.0–4.1

BF model with manufacturing strains

The three samples of each bundle configuration produced a standard deviation of up to 0.27 N (for the peak force of Ring 3) during the experimental testing. This variation can be associated with small dimensional discrepancies of the samples (referring to the ring bundle diameter in particular) and the non-standardized way of tying the turns of each bundle together. Nevertheless, the standard deviation reduced after cycling (e.g., down to 0.078 N for Ring 3, in the region of interest) and the FEA predictions closely matched the experimental result regime. The model predictions for Ring 3 and 4 are inside the standard deviation margin of the experiments while the predictions for Ring 1 and 2 differ by 0.6% and 1.1% from the upper limit, respectively.

These discrepancies were correlated to the diameter of the wire; the thinner the wire, the greater the difference from the experimental values. The fact that the wire diameter greatly affects the results comes as no surprise since the testing evokes primarily bending and it is well known that the radius of a wire dominates its bending stiffness. Moreover, it is interesting to note that in all cases the BF model is stiffer than the experimental bundle; hence, the reported values can infer a force overestimation resulting from the strict confinement of the rotational degrees of freedom at the peaks and valleys of the ring bundle.

Lastly, it is worth mentioning that the analysis time of the saddle-pull validation was less than 4 min per case.

### The Manufacturing Process Effect

In the case where the manufacturing process of wire bending has not been taken into account, the structure is stiffer leading to significantly poorer results (Fig. 7b and Table 6). The reason for this is that before the pulling forces are applied, the material of the bundle is in a zero stress–strain state and as a result can undertake greater strains before entering Nitinol’s plateau (for example Fig. 7b suggests that Ring 4 has not yet entered the martensitic region since no hysteresis is observed). Since, during pulling, the bundle spends more time in the elastic region, it can exhibit greater stiffness when compared to the superelastic response the pre-strained ring bundle presents. It is noted that for the material parameters being used herein, the strain limit of the linear region of austenite Nitinol for 37 °C is 1.08%.

**Table 6:** Deviation of the FEA force–displacement response from the corresponding experimental results for each bundle at its operational region.

Ring	FEA deviation in force from the experimental mean values (%)
Ring 1	22.7–26.3
Ring 2	16.8–22.8
Ring 3	17.0–20.0
Ring 4	14.0–18.8

BF model with no manufacturing strains

### Vascular Deployment: Comparison of the BF and CQ Models

**Figure 8:**
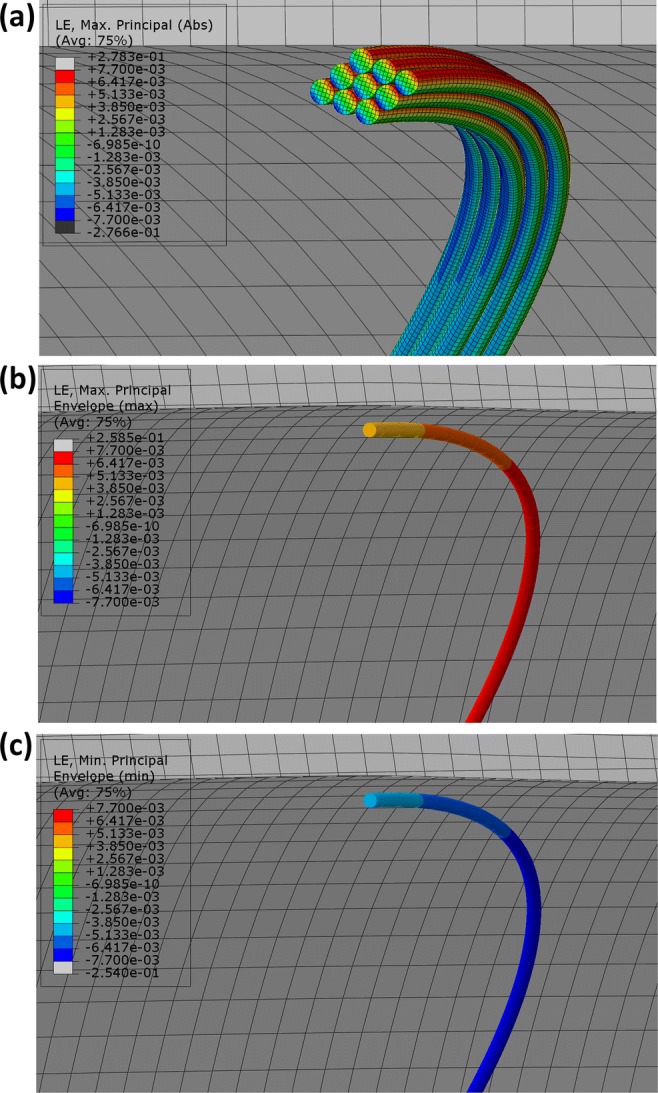
Each turn of the CQ model experiences tension and compression (a). Similarly, all overlapping turns of the BF model have some integration points under tension (b) and some under compression (c).

In the Vascular Deployment study, the BF model and the higher fidelity CQ model were compared. As analysis showed, the individual turns of the CQ bundle acted separately,since each one experienced both tension and compression due to bending (Fig. 8a), rather than some turns (outer) being in complete tension and others (inner) being in complete compression. Likewise, the overlapping turns of the BF model experienced a very similar strain state to the distributed turns of the CQ model (Figs. 8b and 8c); i.e., some section points of the beam elements of each turn were under tension and some were under compression. As a result, each overlapping turn of the BF model underwent a similar mechanical state to any distributed turn of the CQ model, supporting the modeling choice of superimposing the Nitinol wires (illustrated in Fig. 3d).

To quantify the difference between the modeling approaches, the % variation between the turn(s) of the CQ model and the BF model were measured on the parameters of interest; we call this quantity *Var* (the baseline is considered to be the CQ model). For the 1-ring cases, *Var* was a single value, but for the multi-turn cases it was an array, and averaging was conducted $(\overline{Var} )$. The mean and standard deviation of the 4 ring cases were calculated either on *Var* or on $\overline{Var}$ and results are reported in Table 7.

**Table 7:** Mean ± SD of the % variation between the turn(s) of the CQ model and the BF model.

Variables	1-turn model	Multi-turn model
Maximum *ε*_Diast_	− 2.2 ± 1.1	− 5.3 ± 2.7
Maximum *ε*_Syst_	− 0.9 ± 1.6	− 3.4 ± 2.5
Maximum *Mε*	− 3.2 ± 1.1	− 5.2 ± 1.9
COF	0.0 ± 1.1	2.2 ± 1.1

Negative values signify BF model’s underestimation when compared to the CQ model. For the 1-turn model, Ring 4 has been excluded from the results

As can be seen in the 1-turn cases, there is a small underestimation of the BF model prediction of the maximum $\varepsilon_{\text{Diast}}$, $\varepsilon_{\text{Syst}}$ and ${\rm M}\varepsilon$ in the order of 1–3% when compared to the CQ model’s results. For the multi-turn cases, the standard deviation of the results increases as expected, yet not significantly.

Regarding the BF model, strains in the deployed state were higher in the 1-turn versions because the ring was less stiff, hence it deployed less; for the CQ model though, the opposite was observed: the multi-turn cases of the CQ model produced higher maximum *ε* than their respective 1-turn models probably because their turns are distributed, i.e., the turn with the highest curvature in the multi-turn case is under more severe bending than the turn of the 1-turn versions. Note that in all cases, the maximum strains were developed in the peaks or valleys and were in the range of 0.7–1%.

The COF was the variable with the least variation between the modeling methods, having no practical difference in the 1-turn cases and varying by 2.2% at the multi-turn ones. And this, despite the unavoidable difference in the second moment of area between the two models.

Lastly, the analysis time of the BF model was between 180 to 400 minutes depending on the vessel size. The runtime for the 1-turn cases slightly favored the CQ model. However, in the multi-turn cases, the CQ model was 7–23 times slower than the BF. Furthermore, it should be noted that the addition of turns in the BF model did not add any measurable computational cost; on the contrary, runtime for most cases was reduced when the number of turns increased thanks to a stiffer, and as a result more stable, ring. This suggests that the model is very adequate for scaling, i.e., for the simulation of full devices.

## Discussion

Although FEA has become a major tool for stent analysis, the studies conducted in the literature very often employ significant simplifications and sometimes omit comparisons against experimental results, making the conclusions less robust. This, in combination with the fact that the stent type significantly affects the structural response of the device,^7^ has triggered the in-depth study presented here.

In recent investigations, when ring bundles were to be modeled, researchers often adopted the ‘equivalent beam’ approach.^8^,^23^,^24^ This technique assumes that a multi-turn bundle can be represented by a 1-turn model as long as the 2nd moment of area for both of them is the same. Although its simplicity is tempting, the drawback of this approach is significant since such a model exaggerates material strain magnitudes because the radius of the wire is increased. Furthermore, this leads to the bending stiffness of the bundle only being correctly represented at low linear elastic deflections; since Nitinol is non-linear, in high deflections, the equivalent structure will inevitably behave differently from the true structure due to the false stress/strain levels developed. As a result, altering the wire thickness will affect both the strain and the stiffness of the structure making the model inadequate for most structural analyses.

Additionally, these models disregard the manufacturing process of the device, considering the undeployed configuration of the bundle to be unstressed. This strategy was proven herein to add significant errors into the results, not only for strain but for radial stiffness as well. As illustrated, the exclusion of the initial load step leads to high divergences from the saddle-pull experiments in the order of 26%.

In the works of McCummiskey^19^ and Van Zyl,^26^ manufacturing strains have been taken into account on a 1-turn model. These works were important because they captured the mechanical state of the ring more reliably and they allowed for accurate multi-turn analyses to be realized later on by Bow.^4^ Nevertheless, all these studies utilized the double symmetry of the ring, essentially producing quarter ring models, which could not be scaled into full rings for the study of complex anatomies. In addition, these models have necessarily symmetrical constraints because of their construction, thus being inadequate for asymmetric deployments. Furthermore, the use of continuum elements made these approaches computationally demanding, sometimes requiring a full day for a simulation to run on a desktop workstation; a challenging time-frame for research and development use.

Contrary to the above, the BF model developed herein captures the manufacturing strains in a full ring model, does not alter the wire thickness and, in agreement to the physical bundle, has a ‘weld’ connection to link the two ends of the wire. These, in combination with the computational efficiency exhibited, make the model adequate for multi-ring (and full device) scaling and use in asymmetric patient-specific vessel geometries, without altering the fundamental basis of the technique.

Regarding validation, the structural response of the ring stent in isolation was the first variable to be considered. In the literature, different experiments have been used for this parameter in the form of the radial resistive force (RRF) or COF. Gong *et al*.^11^ used a crimping loop both in a laboratory and FEA environment, De Bock *et al*.^6^ used flat plate compression and radial compression tests, while Pelton *et al*.^21^ used a single-strut compression set-up to establish the overall radial compression. Nevertheless, in this study, an alternative—experimental configuration was deemed more appropriate because of the uniqueness of the ring bundle geometry that can easily lead to instabilities in physical testing. More specifically, the uniaxial cycling of the testing machine was transformed into radial deformation for the tested rings; this deformation represented the pulsatile movement of the endograft inside the aorta. As a result, the experiment offered an insight into the overall structural stiffness of the bundle in its operational state, allowing the general response of the structure to be quantified. In addition, this test had the advantage of being isolated from the effects of any radial contact, hence, allowing the bending process within the bundle to be independently evaluated without contact friction effects, something not true for the majority of other radial force testing approaches.

The comparison of four physical rings with their equivalent FEA models revealed similar force–displacement responses while the discrepancies from the experiment always appeared as force overestimations. This effect reflects the exclusion of multiple aspects of the physical bundle such as the wire-to-wire interactions, the change of wire positioning along the length of the bundle, the assumption that the center of mass of the strand always lies at the center of the cross-section of the bundle and the use of a constant value for the stiffness of the polyethylene strings despite the slightly non-linear response produced during the uniaxial testing of the material. Most importantly though, the discrepancies reflect the replacement of friction with total restriction of rotation along the wire’s tangent direction (at the connection points of peaks and valleys) and hence account for the inability to impose the real boundary conditions in every detail. It can be postulated that it is from this overestimation of the friction constraint that the model consistently appears stiffer. Nevertheless, the model produces results inside the standard deviation of the experiment or very close to this range.

When examining key outputs of strain and radial force during the Vascular Deployment study, the CQ and the BF models produced very similar results, in the range of 0–5%, despite their inherent differences. Particularly the discrepancy of radial force was so low that, in accordance with previous studies on different stent designs,^6^,^13^ it can be concluded that beam and continuum elements are equally capable of predicting the COF of ring stent grafts. Unfortunately, no experimental values are available regarding the strains the ring bundle exhibits, particularly because of practical difficulties in taking measurements on such fine wires. Despite the fact that the true value of these variables is unknown though, the small variation between the two models provides confidence for the range in which the results lay.

The BF model developed herein has been shown to be cost effective. The solution time of minutes needed for the saddle-pull test and several hours for the full ring bundle’s compaction, deployment and cycling through systole and diastole is deemed to be low enough to allow a viable, accurate tool for the ring bundle assessment. The respective CQ model, for the same analysis, took anywhere between 7 and 23 times longer to produce results. In their analysis, Hall *et al*.^13^ reported that factor to be 15, making their continuum model lie in the middle of the current results. Direct runtime comparison with other studies in the literature is not trivial since differences in stent geometries, material parameters, the amount of catheter compaction as well as possible exclusion of the cycling steps will inevitably affect the computational cost; reported times from hours to days though can be found in the bibliography.^9^,^24^

Besides the benefits discussed, it is important to mention that some limitations exist. Since the BF model overlaps all Nitinol turns, identification of differences along the width of the bundle is not possible, restricting the capability for a turn-to-turn analysis. Moreover, the circular shape of the bundle is only an approximation of the cross section of the real bundle ring. If detailed contact conditions between the ring and the vessel are of interest (e.g., for direct endoleak assessment) then this approximation might raise difficulties. Lastly, in the vascular deployment, the strain field of the wires was not validated against experimental results. Although such an approach is, in principle, favorable, unfortunately the measuring difficulties that arise due to the size of the wires (0.16–0.22 mm in thickness) and their three-dimensional post-deployment twist did not allow us to pursue it.

In conclusion, a full ring stent bundle has been modeled with a combination of beam and surface finite elements. With the approach presented herein, the shape, the global stiffness, the exerted forces and the strains of the structure can all be well predicted, allowing future studies on stent fatigue and endoleaking to be pursued. At the same time, the overlapping Nitinol turns do not require computationally demanding wire-to-wire interactions to be modeled. This allows design optimization studies to be carried out effectively and paves the way for a full and efficient stent-device model to be implemented within patient-specific applications. This paper, provides the basis of a modeling platform which gives a high degree of confidence in designing for challenging or patient-specific situations that can aid reducing EVAR complications.
